# Clinical study on the combined use of cardiovascular agents and statins in the treatment of coronary heart disease

**DOI:** 10.12669/pjms.42.9.16299

**Published:** 2026-09

**Authors:** Zhili Chen, Qianqian Zhang, Yue Zhang, Zhe Xu, Xiaozhe Yang

**Affiliations:** 1 ZhiLi Chen Department of Cardiology, Shijiazhuang Eighth Hospital, Shijiazhuang 050000, Hebei, China; 2 Qianqian Zhang Department of 1^st^ Cardiology, The No.4 People’ Hospital of Hengshui, Orthopedics Department Hospital, Hengshui 053000, Hebei, China; 3 Yue Zhang Department of 2^nd^ Cardiology, The No.4 People’ Hospital of Hengshui, Orthopedics Department Hospital, Hengshui 053000, Hebei, China; 4 Zhe Xu Department of Pharmacy, The 3th People’s Hospital of Hengshui, Hengshui 053000, Hebei, China; 5 Xiaozhe Yang Department of Clinical Laboratory, The 3th People’s Hospital of Hengshui, Hengshui 053000, Hebei, China

**Keywords:** Cardiovascular agent, Coronary heart disease, Statins, Treatment

## Abstract

**Objective:**

To evaluate the clinical efficacy of combining cardiovascular agents with statins in the treatment of coronary heart disease(CHD).

**Methodology:**

This was a retrospective study. A total of 110 patients diagnosed with CHD and treated at Shijiazhuang Eighth Hospital between January 2024 to December 2025 were enrolled and randomly divided into two groups (n=55 in each group). The control group received conventional therapy, including anticoagulation, coronary vasodilation, antiplatelet therapy, and treatments aimed at relieving myocardial ischemia. The treatment group received atorvastatin in addition to the conventional therapy administered to the control group. Clinical efficacy, lipid profiles, inflammatory markers, adverse reaction rates, and angina episodes were compared between the two groups following treatment.

**Results:**

The overall response rate was 95% in the treatment group and 80% in the control group, with a statistically significant difference(p= 0.02). Post-treatment levels of total cholesterol, triglycerides, and low-density lipoprotein cholesterol were significantly lower in the treatment group than in the control group, while high-density lipoprotein cholesterol levels were significantly higher (p= 0.00, respectively). Inflammatory markers, including interleukin-6, C-reactive protein, and tumor necrosis factor-alpha, were also significantly reduced in the treatment group compared with the control group (p= 0.00). The adverse reaction rate was 13% in the treatment group and 15% in the control group, showing no statistically significant difference(p= 0.76).

**Conclusion:**

The combination of cardiovascular agents with statins, particularly atorvastatin, demonstrates significant clinical benefits in the management of CHD. This regimen can effectively improve lipid metabolism, reduce systemic inflammation, and alleviate anginal symptoms, without increasing the risk of adverse events.

## INTRODUCTION

In recent years, with the rapid advancement of socioeconomic development, the overall quality of life has significantly improved. However, these changes have also led to notable shifts in dietary patterns and lifestyle habits. Unhealthy lifestyles and imbalanced diets have contributed to a rising incidence of cardiovascular diseases.^1^ Coronary heart disease (CHD), also known as coronary atherosclerotic heart disease, is one of the most prevalent cardiovascular conditions. Its primary pathological basis lies in atherosclerosis of the coronary arteries, which leads to luminal narrowing and subsequent functional impairment of coronary circulation. Clinically, this manifests as myocardial hypoxia, ischemia, and, in severe cases, infarction.^2^

Patients with CHD often present with symptoms such as arrhythmias, angina pectoris, and myocardial infarction. Angina associated with CHD typically occurs during physical exertion, resulting from an increased myocardial workload. This significantly elevates the risk of acute cardiac events and is associated with high morbidity and mortality rates.^3^ Notably, early-stage CHD is frequently asymptomatic, which can lead to delayed diagnosis and treatment, posing a serious threat to patient survival. Therefore, timely diagnosis and effective intervention are critical not only for improving patient outcomes and survival but also for preventing complications like heart failure.

Previous studies^4^ have indicated that the onset and progression of CHD are influenced by multiple factors, such as environmental exposures, dietary habits, diabetes, and hypertension. Additionally, smoking and dyslipidemia are recognized as major risk factors.^5^ In clinical practice, pharmacological therapy is the mainstay of treatment for CHD. Particularly, statins are the first-line agents for the treatment of dyslipidemia. Statins, also known as inhibitors of 3-hydroxy-3-methylglutaryl-coenzyme A (HMG-CoA) reductase, reduce the synthesis of endogenous mevalonate and cholesterol and exhibit anti-inflammatory activity, thereby achieving cardiovascular protection.^6^ To further evaluate the therapeutic efficacy of statins in CHD, we conducted a clinical study investigating the combined use of cardiovascular agents and statins. The study yielded promising results.

## METHODOLOGY

This was a retrospective study. A total of 110 patients with confirmed CHD admitted to Shijiazhuang Eighth Hospital between January 2024 and December 2025 were enrolled in this study. Patients were randomly assigned to either the treatment group or the control group, with 55 patients in each group.

### Ethical approval:

The study was approved by the Institutional Ethics Committee of Shijiazhuang Eighth Hospital (No.:2025-10-01; Approval Date: October 1, 2025), and written informed consent was obtained from all participants.

### Inclusion criteria:

- Patients met the clinical diagnostic criteria for CHD^7^, confirmed by coronary angiography and transthoracic echocardiography.
- The patient and his/her family provided informed consent to participate in the study.
- Complete clinical data were available.
- Patients presented with typical clinical symptoms of CHD upon admission, such as palpitations, chest tightness, or chest pain.

### Exclusion criteria:

- Severe dysfunction of the heart, liver, or lungs.
- Patients with severe abnormalities in coagulation function.
- Known hypersensitivity to any of the study drugs.
- History of coronary artery stent implantation.
- Presence of malignancy.
- Pregnant or breastfeeding women.
- Coexisting cardiac diseases, heart failure, or myocardial infarction.
- Cognitive impairment or psychiatric disorders.

### Treatment methods:

On the first day after admission, patients in the control group received standard therapy, which included symptomatic treatments such as anticoagulation, coronary vasodilation, antiplatelet aggregation, and improvement of myocardial ischemia. Commonly used medications included aspirin, low-molecular-weight heparin, and isosorbide mononitrate. In addition, comorbid conditions were managed accordingly. Patients with coexisting hypertension were treated with antihypertensive agents; those with diabetes received insulin or oral hypoglycemic agents; and patients with pulmonary dysfunction were given appropriate anti-infective therapy and interventions to improve respiratory function. The treatment group received atorvastatin in addition to the conventional therapy administered to the control group. Atorvastatin was prescribed at a dosage of 20 mg once daily, taken orally in the evening, for a duration of three months. During the treatment period, hepatic and renal function were closely monitored in both groups to ensure medication safety. The follow-up time for patients in both groups was three months.

### Clinical efficacy assessment:

Treatment efficacy was evaluated in both groups after three months and classified as follows:

### Complete Response (SR):

Clinical symptoms were nearly resolved, and the frequency of angina attacks was reduced by more than 80% compared with baseline.

### Partial Response (PR):

Clinical symptoms were significantly improved, and the frequency of angina attacks was reduced by 50% to 80% compared with baseline.

### No Response (NR):

Clinical symptoms showed no improvement or worsened, and the frequency of angina attacks did not meet the above criteria. Overall response rate (ORR)= (SR cases + PR cases) / Total number of patients × 100%.

### Lipid profile:

Fasting venous blood samples were collected from both groups before and after treatment. Serum levels of total cholesterol (TC), triglycerides(TG), low-density lipoprotein cholesterol (LDL-C), and high-density lipoprotein cholesterol (HDL-C) were measured using an automated biochemical analyzer. Pre- and post-treatment values were compared within and between groups.

### Inflammatory markers:

Fasting morning venous blood was drawn from all patients before and after treatment. C-reactive protein (CRP) levels were determined using immunoturbidimetry, while interleukin-6 (IL-6) and tumor necrosis factor-alpha (TNF-α) levels were measured using enzyme-linked immunosorbent assay (ELISA).

### Adverse reactions:

Adverse events occurring during the treatment period were documented in both groups, including diarrhea, vomiting, and headache.

### Angina assessment:

Post-treatment angina episodes were evaluated based on the frequency of attacks, pain intensity, and duration. Pain intensity was assessed using the Visual Analogue Scale (VAS), with scores ranging from 0 (no pain) to 10 (most severe pain). Higher scores represent greater pain intensity.

### Statistical analysis:

All statistical analyses were performed using SPSS 20.0. Measurement data were expressed as mean ± standard deviation (*x̅*±*s*), and subjected to Shapiro-Wilk test and followed a normal distribution. Between-group comparisons were conducted using the independent samples t-test, while within-group comparisons were performed using the paired t-test. Comparisons of percentages were analyzed using the chi-square (χ²) test. A *p*-value of <0.05 was considered statistically significant.

## RESULTS

The treatment group consisted of 35 males and 20 females, aged 50–72 years (mean age: 62.14 ± 6.23 years), with disease duration ranging from three to seven years (mean duration: 5.62 ± 1.85 years). The control group included 31 males and 24 females, aged 46–72 years (mean age: 61.48 ± 6.31 years), with disease duration between four and seven years (mean duration: 6.08 ± 2.27 years). There were no statistically significant differences in baseline characteristics between the two groups, indicating comparability (Table-I).

**Table-I:** Comparison of baseline characteristics between the treatment and control groups (*x̅*±*s*, n = 55).

Item	Treatment	Control	t/χ²	p
Age (years)	62.14±6.23	61.48±6.31	0.52	0.58
Male (n[%])	35 (%)	31 (%)	0.61	0.44
Disease duration (years)	5.62±1.85	6.08±2.27	1.16	0.25
***Past history***				
Hypertension	12(%)	13(%)	0.05	0.82
Diabetes	8(%)	6(%)	0.32	0.57
Hyperlipidemia	10(%)	11(%)	0.06	0.81
Type of angina pectoris			0.60	0.43
Stable	34(%)	30(%)		
Unstable	21(%)	25(%)		
***Degree of coronary stenosis***				
Mild	20(%)	17(%)	0.37	0.55
Moderate	26(%)	27(%)	0.04	0.85
Severe	9(%)	11(%)	0.24	0.62

p > 0.05.

The ORR was 95% in the treatment group and 80% in the control group. The treatment group exhibited a significantly higher ORR than the control group (p= 0.02) (Table-II).

**Table-II:** Comparison of clinical efficacy between groups (n = 55, *x̅*±*s*).

Group	SR	PR	NR	ORR
Treatment	30	22	3	52 (95%)
Control	27	17	11	44 (80%)
*χ^2^*				5.24
*p*				0.02

p < 0.05.

No significant differences were observed in serum TC, TG, LDL-C, and HDL-C levels between the two groups before treatment (*p >* 0.05). However, after three months of treatment, the treatment group showed significantly lower levels of TC, TG, and LDL-C, and significantly higher HDL-C levels compared with the control group (all *p=* 0.00) (Table-III).

**Table-III:** Comparison of pre- and post-treatment lipid profiles between groups (n = 55, *x̅*±*s*).

Item		Treatment	Control	t-value	p-value
TC (mmol/L)	Pre-treatment	6.92±0.37	7.03±0.45	1.41	0.16
	Post-treatment*	4.36±0.22	5.16±0.34	10.65	0.00
TG (mmol/L)	Pre-treatment	2.36±0.45	2.33±0.30	0.40	0.69
	Post-treatment*	1.05±0.21	1.68±0.42	9.95	0.00
LDL-C (mmol/L)	Pre-treatment	3.63±0.87	3.70±0.76	0.45	0.65
	Post-treatment*	1.64±0.47	2.23±0.62	5.62	0.00
HDL-C (mmol/L)	Pre-treatment	1.20±0.20	1.17±0.18	0.83	0.41
	Post-treatment*	1.76±0.33	1.30±0.25	8.24	0.00

p < 0.05.

Changes in serum inflammatory markers before and after treatment are shown in Table-IV. There were no significant differences in baseline levels of IL-6, CRP, and TNF-α between the two groups (all *p >* 0.05). After treatment, all three inflammatory markers were significantly lower in the treatment group compared with the control group (*p=* 0.00).

**Table-IV:** Comparison of pre- and post-treatment inflammatory marker levels between groups (n = 55, *x̅*±*s*).

Marker	Time Point	Treatment	Control	t-value	p-value
IL-6 (ng/L)	Pre-treatment	13.73±5.27	3.46±5.18	0.31	0.62
	Post-treatment*	8.61±2.31	10.57±2.92	4.01	0.00
CRP (mg/L)	Pre-treatment	4.31±0.85	4.28±0.79	4.57	0.54
	Post-treatment*	2.28±0.14	2.58±0.39	6.28	0.00
TNF-α (ng/L)a	Pre-treatment	43.46±12.81	43.60±12.54	0.32	0.73
	Post-treatment*	23.31±7.84	26.70±8.58	5.21	0.00

* p < 0.05.

The ARR was 13% in the treatment group and 15% in the control group, indicating no statistically significant difference between the two groups (*p =* 0.76) (Table-V).

**Table-V:** Comparison of ARRs between groups (n = 55, *x̅*±*s*).

Group	Dizziness/Headache	Gastrointestinal Reactions	Insomnia	Overall ARR*
Treatment	3	2	2	7(13%)
Control	4	3	1	8(15%)
*χ^2^*				0.08
*p-value*				0.76

p > 0.05.

After treatment, the treatment group showed significantly fewer weekly angina attacks, lower pain intensity (VAS score), and shorter duration of symptoms compared with the control group (all *p=* 0.00) (Table-VI).

**Table-VI:** Comparison of post-treatment angina outcomes between groups (n = 55, *x̅*±*s*).

Group	Frequency of Attacks (episodes/week)*	Pain Intensity (VAS score)*	Duration of Symptoms (min)*
Treatment	1.87±0.24	2.31±0.12	1.53±0.11
Control	3.26±0.59	3.06±0.32	2.23±0.20
*t-value*	25.77	16.27	15.10
*p-value*	0.00	0.00	0.00

* p < 0.05.

## DISCUSSION

In this study, the treatment group receiving combination therapy with atorvastatin achieved an ORR of 95%, significantly higher than 80% in the control group (p = 0.02). After treatment, the treatment group showed significantly lower levels of TC, TG, and LDL-C, and significantly higher HDL-C levels than the control group (all p = 0.00). Additionally, patients in the treatment group exhibited fewer weekly angina episodes, lower VAS scores, and shorter symptom duration (p = 0.00, respectively). These findings may be partly attributed to the fact that many patients with CHD exhibit poor dietary habits, including high intake of carbonated beverages and fatty foods, which contribute to elevated blood lipid levels. Previous research has demonstrated that each 1 mmol/L reduction in serum LDL-C is associated with a 10%–20% decrease in the incidence of cardiovascular events, while reductions of 2–3 mmol/L can yield risk reductions of up to 50%.^8^ These data underscore the vital importance of aggressive lipid-lowering in the treatment of CHD and the prevention of adverse cardiovascular events. Moreover, atorvastatin has also been shown to elevate HDL-C and reduce TG levels to a certain extent,^9^ consistent with the findings of our study.

Dyslipidemia is a major risk factor for the development of CHD. Elevated lipid levels not only damage vascular endothelial cells and trigger atherosclerosis but also induce systemic inflammatory responses, which further aggravate endothelial injury and accelerate thrombus formation. Ridker PM et al.^10^ have demonstrated a strong association between inflammatory cytokines (e.g., IL-6, CRP, TNF-α) and the progression of CHD. Among these, CRP is widely recognized as a key biomarker for assessing systemic inflammation in cardiovascular disease, while IL-6 plays an active role in the thrombogenic process. TNF-α, with its pleiotropic biological effects (e.g., antimicrobial, antitumor, and antiviral activity), is also involved in the pathogenesis of cachexia and cardiovascular disorders.

Evidence has confirmed a positive correlation between hyperlipidemia and the incidence of CHD, highlighting the critical importance of dyslipidemia prevention and control in patients with CHD.^11^-^13^ In CHD, atherosclerosis leads to endothelial injury and dysfunction, promoting platelet activation and aggregation, which in turn results in thrombus formation.^14^ Hyperlipidemia not only exacerbates disease progression but also significantly increases the risk of adverse cardiovascular events, posing a serious threat to patient survival and greatly impairing quality of life. Existing literature also suggests that elevated levels of reactive oxygen species and the associated lipid peroxidation may contribute to the pathogenesis of atherosclerosis.

These oxidative processes can damage vascular smooth muscle and endothelial cells, leading to altered cellular function, upregulation of adhesion molecules, and infiltration of lipids into the intima. Macrophages then internalize oxidized low-density lipoproteins, thereby accelerating apoptosis of smooth muscle cells. As a result, lipid accumulation and plaque formation occur, advancing coronary atherosclerosis.^15^ Furthermore, lipid accumulation, oxidative stress, and inflammatory responses are interrelated and reinforce one another, creating a vicious cycle that perpetuates vascular injury and plaque progression. Therefore, in the clinical management of patients with CHD and hyperlipidemia, aggressive lipid-lowering therapy is essential to disrupt this pathological cycle.

Statins are lipid-regulating agents classified as selective inhibitors of HMG-CoA reductase.^16^ Atorvastatin, in particular, inhibits the endogenous synthesis of cholesterol and effectively regulates lipid metabolism, thereby slowing disease progression in patients with CHD. In individuals with CHD complicated by hyperlipidemia, excess lipids in the blood tend to accumulate in the intimal layer of arteries, forming atheromatous plaques that progressively narrow the vascular lumen. This impedes blood flow and leads to myocardial ischemia, with angina pectoris being a common clinical manifestation that severely impairs patients’ quality of life.^17^ The use of statins in patients with CHD and dyslipidemia plays a crucial role in halting or slowing the progression of atherosclerosis, preventing the development of new lesions or total occlusion, and significantly reducing the risk of myocardial infarction and cardiovascular mortality.

A large body of epidemiological research has confirmed that high-sensitivity CRP (hs-CRP) and IL-6 levels are closely associated with the risk of cardiovascular events.^18^ Accordingly, hs-CRP is now commonly employed as a critical marker for evaluating inflammation in patients with cardiovascular disease. In our study, patients receiving combination therapy with statins exhibited significantly lower post-treatment levels of IL-6, CRP, and TNF-α compared with those in the control group (p = 0.00, respectively), confirming the potent anti-inflammatory effects of atorvastatin. Similar findings were reported by Hao Y et al.^19^, who observed that treatment with atorvastatin significantly reduced serum levels of CRP, IL-6, and TNF-α compared with baseline and untreated controls. Engebretsen I et al.^20^ also proposed that atorvastatin may inhibit the activity of matrix metalloproteinases and intercellular adhesion molecule-1, thereby preventing the aggregation and chemotaxis of inflammatory cells within coronary plaques. This mechanism contributes to the alleviation of inflammation and the suppression of atherosclerotic progression.

From the safety perspective, ARRs in the treatment group did not significantly increase compared with the control group (all p >0.05). Although statins have been associated with potential hepatotoxicity and elevated transaminase levels, no clinically significant liver dysfunction was observed in the treatment group during the study period. The relatively low ARRs might be attributed to the short duration of statin administration.

### Limitations:

Despite all this, the current study still has several limitations. First, the follow-up duration was relatively short, and the sample size was small. Second, the study did not include other types of statins for horizontal comparison, which may have restricted the comprehensiveness of the efficacy and safety evaluation.

## CONCLUSIONS

In short, the combination of statins with conventional cardiovascular agents demonstrates significant therapeutic benefits in patients with CHD. This treatment regimen can effectively improve lipid profiles and relieve systemic inflammation, while also reducing the frequency, intensity, and duration of angina episodes. Importantly, the addition of statins did not lead to a significant increase in adverse reactions, indicating a favorable safety profile and promising clinical applicability.

### Recommendations:

Future research should involve larger, multicenter cohorts with extended follow-up periods and more objective, scientific study designs. These efforts may help establish a more comprehensive understanding of the benefits and risks of statin-based combination therapies in CHD management, ultimately improving outcomes for a broader patient population.

### Authors’ Contributions:

**ZC** and **QZ:** Designed this study, prepared this manuscript, and are responsible and accountable for the accuracy or integrity of the work.

**YZ, ZX** and **XY:** Collected, and analyzed clinical data. Critical Review.

All authors have read and approved the final manuscript.
